# Analysis of the regulation networks in grapevine reveals response to waterlogging stress and candidate gene-marker selection for damage severity

**DOI:** 10.1098/rsos.172253

**Published:** 2018-06-27

**Authors:** Xudong Zhu, Xiaopeng Li, Songtao Jiu, Kekun Zhang, Chen Wang, Jinggui Fang

**Affiliations:** Nanjing Agricultural University, Nanjing 210095, People's Republic of China

**Keywords:** grapevine, waterlogging, transcriptome, gene expression, marker gene

## Abstract

Owing to the climate change impacts, waterlogging is one of the most hazardous abiotic stresses to crops, which also can result in a serious reduction in the quantity and quality of grape berry and wine production during the rainy season. Therefore, the exploration of the response mechanism of grape to waterlogging is necessary, for which the analysis of the transcriptomic regulation networks of grapevine leaves in response to waterlogging stress was carried out. In this study, 12 634 genes were detected in both waterlogging stress and control grapevine plants, out of which 6837 genes were differentially expressed. A comparative analysis revealed that genes functioning in the antioxidant system, glycolysis and fermentation pathway, chlorophyll metabolism, amino acid metabolism and hormones were activated to reduce injury to grapes under the waterlogging stress. Meanwhile, genes encoding class-2 non-symbiotic haemoglobin were determined as important in waterlogging acclimation. Additionally, the expression variations of three marker genes were found to be informative and can be used to predict the viability of the grapevines subjected to waterlogging. This research not only probes the molecular mechanism underlying grapevine waterlogging tolerance but also puts forward an idea about the application of gene expression information to practical management.

## 1. Introduction

Grape (*Vitis vinifera*) cultivation has spread to all parts of the world. The grape industry in China has developed rapidly in recent years, and China had the world's largest total grape production and the world's second largest vineyard in 2015, based on the OIV statistical report on the world vitiviniculture situation in 2016. However, China's table and wine grape industry is now encountering a thorny problem. Located on the western shore of the Pacific Ocean, most parts of China are dominated by a continental monsoonal climate, characterized by extreme weather events such as rainy summer–autumns. Heavy rains that often result in flooding or waterlogging are very detrimental to grapevines, especially during the rainy season, commonly called the plum rain season, which continues nearly two months during the late spring and early summer in the southern part of China. Grape production, wine quality and importantly flavour or ‘style' distinguish wine from other agricultural products that are very dependent upon climate. Heavy rains and bad climatic conditions affect grape growth and berry development. They are unfavourable for sugar accumulation, organic acid degradation and phenolic compound formation, which seriously hinder the further development of the table and wine grape industry in China.

Waterlogging creates low-oxygen (hypoxic) environments in the root areas that inhibit ATP generation from oxidative phosphorylation [1], which results in a limited supply of energy for nutrient uptake and transport [2]. Finally, waterlogging conditions lead to the induction of an energy-conserving pathway that decreases oxygen consumption, i.e. the metabolic switch from aerobic respiration to anaerobic fermentation, and improves plant performance [3,4]. Moreover, waterlogging also causes significant physiological and morphological changes in plants, such as some reduction in stomatal conductance, photosynthesis and root hydraulic conductivity. These changes affect carbohydrate reserves and translocation [3].

The response of plants to external hypoxia has been intensively studied in the past and field observations showed altered energy metabolism and growth rates, photosynthetic rates and mineral nutrient content in waterlogged plants. Subsequently, molecular investigations and mRNA profiling studies have demonstrated that these adaptive responses occurred at the transcriptome level in *Arabidopsis* [5–7] and crop species such as rice (*Oryza sativa*) [8], cotton (*Gossypium hirsutum*) [1] and poplar (*Populus canescens*) [9]. The low-oxygen environment stress-activated genes encode proteins and enzymes for anaerobic fermentation, glycolysis, transcription factors and signalling pathways in order to allow biological and physiological adjustments to the low-oxygen conditions [10]. All of these rapid changes take place in a large number of transcripts involved in hypoxic or anoxic response, indicating that plants have complex responses to low oxygen.

The studies on plants' responses to waterlogging stress have also been focused on crops such as maize [11], cucumber [12], sesame [13] and rape [14]. However, few reports about the response of grapevine to waterlogging at the molecular level were found. Several published studies reported the physiological responses of grapevine plants to waterlogging stress [15–18]. Field experiments with waterlogged grapevine have recorded that the effects of waterlogging stress include reduction in stomatal conductance, photosynthetic rate and plant height, as well as premature senescence and disturbances to yield components.

Despite these lines of evidence, the molecular mechanism underlying the waterlogging response in grapevine remains unknown. High-throughput transcriptome analysis could offer good help to improve our understanding of the molecular responses to waterlogging in grapevine. To gain insight into the molecular responses of grapevine to waterlogging, here, we profiled transcriptome changes in grapevine aerial leaves subjected to waterlogging using high-throughput sequencing, as well as the physiological responses of seedlings to waterlogging.

The rapid development of gene detection techniques, e.g. one-step polymerase chain reaction (PCR) technique, which is a simple, rapid and sensitive technique in detecting gene expression levels, provides a possibility for us to apply gene transcription data to practical production. As gene expression always occurs prior to the corresponding morphological changes, the information of the gene expression variation trends detected can provide the signal used to predict or pre-diagnose the occurrence of the corresponding phenotype. This is of much significance for crop production, especially in regions where crops suffer greatly from adverse environmental stress such as drought, waterlogging and high salinity. According to this principle, Wang *et al*. [13] depicted the grapevine phenology by analysing the expression profiles of nine grapevine flower and berry development genes and verified the workability of the application of gene expression to depicting grapevine phenology with fertilization trials [19], which followed the same principle that had been applied to pre-diagnose some diseases in humans in the field of medicine [20–22]. In this study, the transcriptomic analysis of the response of grapevine leaves to waterlogging can help us gain deeper insights into the low-oxygen stress-responsive mechanisms in grapevine and facilitate our understanding of the response of flood-tolerant woody plants to soil waterlogging stress. Moreover, the research work on the marker genes that can be used to diagnose the damage from waterlogging to grapevine is a novel and successful trial in exploring the practical use of gene information.

## 2. Material and methods

### 2.1. Plant materials

Two-year-old clonally propagated and virus-free rootstock plants from ‘Summer Black' (hybrids of *V. vinifera* and *V. labrusca*) grapevine were planted in plastic pots containing peat, vermiculite and perlite (3 : 1 : 1, v/v) in a ventilated greenhouse under natural lighting and temperature (28°C/18°C, 12 L : 12 D) and a relative humidity ranging from 70% to 85% at the experimental farm of the Nanjing Agriculture University during the growing season until the waterlogging experiment. Plants were watered three times a week with tap water and fertilized every two weeks with 1 g/pot of N : P : K (25 : 10 : 10).

### 2.2. Waterlogging treatments and recovery assay

Once the plants reached an average height of 60 cm, all the 70 plants were divided into two groups: one served as the control sample (CK) with a total of 10 plants watered regularly to maintain vigorous growth, while the others, totalling 60 plants, were moved to plastic containers and filled with water (pH 7.03, 25°C, electrical conductivity 0.34 dS m^−1^, dissolved oxygen level 7.17 mg l^−1^) to above the soil level so that the soil surface of the pots was covered with a thin layer (4 cm) of water immersed as the waterlogging-treated sample (CT).

The leaves of CT were sampled at 0, 24, 48, 72, 96, 120 and 144 h from randomly selected plants after the application of fresh water. Then the 10 plants selected at each time point were shifted to normal growth conditions, as Recovery treatment, and the survival rate of plants was then calculated.

For the high-throughput sequencing analysis, the fourth leaf from the shoot apex was collected from the three biological replicates of both CK and CT at 48 h of waterlogging treatment, respectively, and the three samples were mixed to make one composite sample.

For the qRT-PCR analysis, the fourth leaf from the shoot apex was also collected from CK and CT at the eight time points (0, 12, 24, 48, 72, 96, 120, 144 h), and was frozen in liquid nitrogen and stored at –80°C.

### 2.3. RNA extraction, cDNA library construction and Illumina deep sequencing

Total RNA samples of CT and CK were extracted using Trizol reagent (Invitrogen, Carlsbad, CA, USA) and subsequently used for mRNA purification and library construction with the Ultra™ RNA Library Prep Kit for Illumina (NEB, USA) following the manufacturer's instructions. The samples were sequenced on an Illumina Hiseq^TM^ 2500. Each sample yielded more than 4 Gb of data. Sequencing was completed by the Shanghai Hanyu Biotechnology Company (Shanghai, China).

### 2.4. Analysis of gene expression level

After adaptor trimming and quality trimming, the clean reads were mapped to the *V. vinifera* transcriptome using Bowtie2 [23]. Then, SAM tools and BamIndexStats.jar were used to calculate the gene expression level, and the RPKM value was computed from SAM files. The gene expression difference between the log and early stationary phase was obtained by MARS (MA-plot-based method with the Random Sampling model), a package from DEGseq [24]. We simply defined genes with at least onefold change between two samples and a false discovery rate (FDR) less than 0.001 as differentially expressed genes (DEGs). Transcripts with |log_2_FC| < 1 were assumed have no change in expression levels.

### 2.5. Transcriptome analysis

To get high-quality clean reads, in-house perl scripts were used to process raw data, which removed reads containing adapters, low-quality reads and reads containing poly-N. The calculation of Q20, Q30, GC-content and sequence duplication level, and other downstream analyses were based on the clean reads. Transcriptome assembly was achieved using Trinity [25]. The *V. vinifera* genome, gene models and annotation of version V2 were downloaded from the Grape Genome Database (http://genomes.cribi.unipd.it/grape/). The genome from *V. vinifera* was used as sequence of reference. Gene function was annotated based on the following databases: KOG/COG (Clusters of Orthologous Groups of proteins), UniProt (a comprehensive resource for protein sequence and annotation data), KO (KEGG Ortholog database) and GO (Gene Ontology), using BLAST with a cut-off E-value of 10^−5^.

### 2.6. Photosynthesis measurements and enzymatic activity assays

The chlorophyll a and b are, respectively, determined by spectrophotometric measurement at 663 and 645 nm. Superoxide dismutase (SOD) activities were determined by monitoring its ability to inhibit photochemical reduction of nitroblue tetrazolium at 560 nm [26]. Peroxidase (POD) activities were determined by using the guaiacol oxidation method [27]. Catalase (CAT) activities were determined by monitoring the disappearance of H_2_O_2_ and by measuring the decrease in absorbance at 240 nm [28]. Three technical repeats were generated for all the quantifications.

Results were expressed as means ± s.e. The SPSS v22 software was used for statistical analysis. To assess the statistical significance of the treatment differences, a one-way analysis of variance (ANOVA) followed by Duncan's multiple range test (with *p* set at 0.05) was employed.

### 2.7. Quantitative real-time polymerase chain reaction analysis

Quantitative real-time (qRT)-PCR analysis was used to verify the DEG results and select a candidate gene marker. The RNA samples used for the qRT-PCR assays were collected as mentioned above. Gene-specific primers were designed according to the reference unigene sequences using Primer Premier 5.0 (electronic supplementary material, table S1). qRT-PCR was carried out using an ABI PRISM 7500 real-time PCR system (Applied Biosystems, USA). SYBR green reaction mix (SYBR® Green qRT-PCR Master Mix, Toyobo, Osaka, Japan) was used in RT-PCR reactions, according to the manufacturer's instructions. For each reaction, 1 µl of diluted cDNA (equivalent to 100 pg total RNA) was mixed with 10 µl 2×SYBR green reaction mix, and 5 pmol of the forward and the reverse primers were added to make a final volume of 20 µl. Each pair of qRT-PCR primers were validated by cloning and sequencing of the RT-PCR product with this pair of primers. The conditions for PCR amplification were as follows: polymerase activation at 95°C for 1 min, followed by 50 cycles of 95°C for 15 s, 95°C for 15 s, 60°C for 20 s and 72°C for 20 s. The fluorescence signal was measured once every one cycle. The *Ct* values were converted into relative copy numbers using a standard curve [29]. Grape *actin* gene rRNA was used as an endogenous control gene in the qPCR detection of miRNAs (forward: 5′- GCATCCCTCAGCACCTTCCA -3′, and reverse: 5′- AACCCCACCTGAACACATCTCC-3′), and the relative expression levels of genes were presented by 2^−Δ*Δ*CT^. Data were analysed with an *R^2^* above 0.998 using the LinRegPCR program [30].

## 3. Results

### 3.1 Morphological and physiological variations in responses to waterlogging and during recovery

Plants of grape cv. ‘Summer Black’ grown in pots were subjected to waterlogging for 144 h. After waterlogging treatment, the waterlogged plants were drained and kept in a greenhouse. The growing status of the plants studied was monitored.

**Figure 1.**
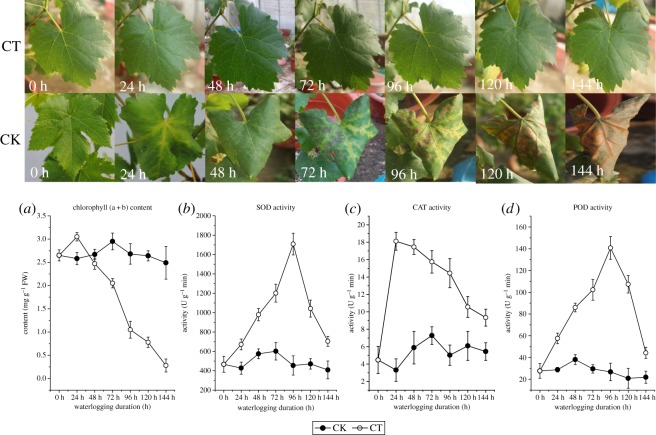
Morphological and physiological changes of leaves in response to waterlogging stress. The observed changes in the leaves of ‘Summer Black' plants that were waterlogged for 0, 24, 48, 72, 96, 120 and 144 h are shown here. The effects of waterlogging on the total chlorophyll (a + b) content (*a*) and on the activity of SOD (*b*), CAT (*c*), POD (*d*) of the leaves of grapevine are also shown here. CK, non-waterlogged; CT, waterlogged for between 0 h and 144 h. Means and standard errors based on three replications are indicated.

Morphological changes of waterlogged leaves were observed. Leaves of the grapevine subjected to flooding gradually wilted, turned yellow or brown, followed by premature senescence and abscission (figure 1). It is obvious that the percentage of leaves that turned yellow and curly significantly increased after the waterlogging treatment (table 1).

**Table 1:** Percentage of the leaves turning yellow and curly.

	0 h	24 h	48 h	72 h	96 h	120 h	144 h
CT	0%	7.7%	19.2%	48.1%	57.7%	73.1%	96.2%
	0/52	4/52	10/52	25/52	30/52	38/52	50/52
CK	0%	0%	0.2%	0.5%	0.7%	0.7%	0.7%
	0/43	0/43	1/43	2/43	3/43	3/43	3/43

To characterize physical symptoms in the leaves of grapevine under waterlogging stress, the content changes of chlorophyll and the activities of stress-related enzymes were further monitored. It is well established that waterlogging stress leads to some physiological changes in waterlogged tree organs, an observation similar to that of the previous studies on the influence of waterlogging on photosynthetic rates and carbohydrate content in flooded plants [31,32]. The mean leaf chlorophyll (a and b) contents are kept stable during the early stages of waterlogging (before 48 h). It was followed by a progressive fall in chlorophyll contents, especially during the period from 72 h to 144 h. After 48 h, the leaf chlorophyll content was lower than that in CK leaves, and reached the lowest level of 11.2% (compared to CK) at 144 h (figure 1*a*).

Overproduction of reactive oxygen species (ROS) and overexpression of various antioxidative enzymes involved in the ROS scavenging system of plants occur almost in all biotic and abiotic stress conditions [33]. In this study, it was found that the activities of SOD, CAT and POD in control grapevine leaves were low and stable, but those in the leaves of the grape plants under waterlogging stress increased substantially, and began declining after 24 h (CAT) and 96 h (SOD, POD) under the stress condition (figure 1*b–d*). SOD and POD activities were significantly higher in waterlogging than those in control plants at 96 h of treatment, while CAT expressed a high level at 24 h. Their highest levels were 3.8-, 5.2- and 2.9-fold those in the control plants, respectively. This could confirm the fact that SOD, CAT and POD played important roles in the ROS scavenging system, while the speed of CAT response to the stress was greater than that of POD and SOD.

All the observed variations in some stress-related organic compound content and enzymes activities were in accordance with the previous reports about the impact of waterlogging stress on grapevine [15–18].

### 3.2. Transcriptome sequencing and assembly

To examine changes in global gene transcription in the leaves of waterlogged grape seedlings, we analysed the transcriptional profile of the leaves of ‘Summer Black’ plants exposed to waterlogging stress for a short period (48 h). Two leaf libraries (non-waterlogging versus waterlogging) were constructed from grape seedlings. A total of 21.2 million and 31.2 million Illumina PE raw reads were generated from the CK and CT libraries, respectively (table 2). After removing adaptor sequences, ambiguous nucleotides and low-quality sequences, there were 20 224 598 and 29 927 131 clean reads remaining from the CK and CT libraries, respectively. From the total reads, 53.83% matched either to a unique (44.79%) or to multiple (9.04%) genomic locations were recorded (table 2). Assembly of clean reads resulted in 42 895 unigenes in the range of 201–14 055 bp with an N50 length of 1028 bp. The majority of the reads could be mapped to the grape genome.

**Table 2:** Summary of sequences analysis. CK, controlled sample; CT, waterlogging-treated sample. Q20: the percentage of bases with a Phred value >20; Q30: the percentage of bases with a Phred value >30; total mapped reads: total reads with at least one reported alignment; unique match: reads with only one reported alignment.

sample	CK	CT
raw reads	21 238 753	31 226 456
clean bases	20 224 598	29 927 131
error (%)	0.05	0.05
Q20 (%)	98.40%	98.31%
Q30 (%)	98.40%	98.31%
GC (%)	98.40%	98.31%
total mapped reads	53.83%	
unique match	44.79%	

### 3.3. Sequence annotation

The unigenes were annotated by aligning with the several public databases (table 3). Analyses showed that 9326 unigenes (21.74%) had significant matches in the GO database, 9032 (21.06%) in the KOG database and 3022 (7.04%) in the KEGG database. In total, 12 634 unigenes (29.45%) were successfully annotated in at least one of the KEGG, GO and KOG databases, with 934 unigenes (2.18%) in all three databases.

**Table 3:** BLAST analysis of non-redundant unigenes against public databases.

	number of unigenes	percentage
annotated in GO	9326	21.74%
annotated in KOG	9032	21.06%
annotated in KEGG	3022	7.04%
annotated in Uniprot	29 971	69.87%
annotated in all databases	934	2.18%
annotated in at least one database	12 634	29.45%
total unigenes	42 895	

**Figure 2.**
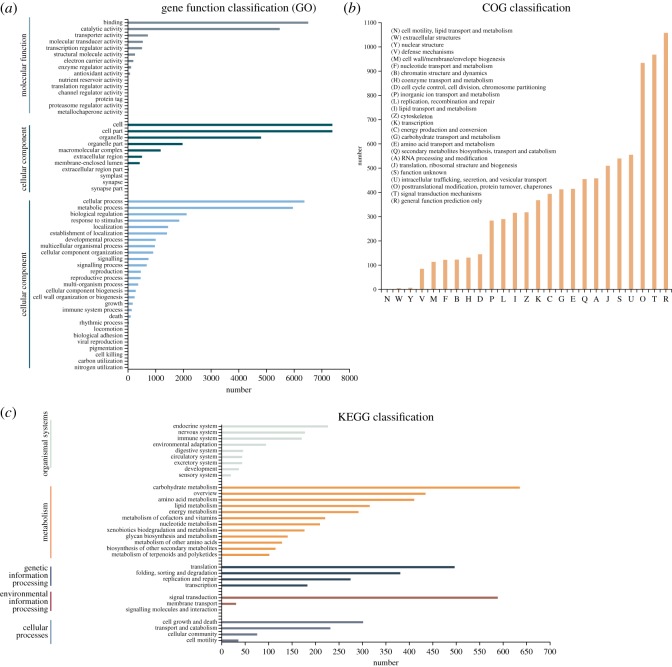
(*a*) GO categorization of non-redundant unigenes. (*b*) COG annotations of putative proteins. (*c*) KEGG annotations of putative proteins.

GO analysis showed that 9326 unigenes could be divided into three ontologies (figure 2*a*). With regard to the biological process (BP) category, the most highly represented genes were associated with ‘cellular process' (6376), ‘metabolic process' (5963) and ‘biological regulation' (2126). The cellular component (CC) category mainly comprised proteins involved in ‘cell’ (7387), ‘cell part' (7387) and ‘organelle' (4812). Within the molecular function (MF) category, ‘binding' (6515), ‘catalytic activity' (5483) and ‘transporter activity' (724) were highly represented.

In addition, all unigenes were subjected to a search against the COG database for functional prediction and classification. In total, 9032 unigenes were assigned to COG classification and divided into 26 specific categories (figure 2*b*). The ‘general functional prediction only' (1059) was the largest group, followed by ‘Signal transduction mechanisms' (969), ‘Posttranslational modification, protein turnover, chaperones' (935), ‘Intracellular trafficking, secretion and vesicular transport' (556) and ‘Function unknown' (541). Only a few unigenes were assigned to ‘Nuclear structure' (8) and ‘Extracellular structures' (6), and no unigenes were assigned to ‘Cell motility, lipid transport and metabolism'.

The unigene metabolic pathway analysis was also conducted using the KEGG annotation system. This process predicted a total of 32 pathways with a total of 3023 unigenes functionally classified (figure 2*c*). The pathways involving the highest number of unique transcripts were ‘translation' (497), followed by ‘carbohydrate metabolism' (395) and ‘Folding, sorting and degradation' (381). The pathways involving the lowest number of unique transcripts were ‘Development' (37), ‘Cell motility' (36), ‘Membrane transport’ (31) and ‘Sensory system' (20). Only one unigene was assigned to the pathway of ‘Signaling molecules and interaction' (1).

### 3.4. Differential expression analysis of transcripts in the leaves of waterlogged seedlings

In this study, a total of 29 971 (69.87% of 42, 895) transcripts in the grapevine leaf were identified, including 20 647 in the CK and 21 542 in the CT library, respectively. Among all the expressed transcripts, the expression levels of 23 584 transcripts showed no significant changes (|log_2_foldchange (log_2_FC)| < 1) and 6837 ones were significantly regulated under waterlogging stress (|log_2_FC)| ≥ 1 with a low FDR <0.001).

Further, rigorous algorithm method was applied to identify DEGs from the normalized data by pairwise comparisons of the waterlogging stress treatment and control samples. The DEGs (q-value < 0.005 and |log2 (foldchange)| > 1) were defined as genes that were significantly enriched or depleted in one tissue relative to the other tissue. To characterize the genes involved in the response to waterlogging in leaves, the expression profiles in the waterlogging treatment (CT) leaves and the control (CK) leaves were compared. Statistical analysis of the frequency of genes identified that 6837 genes showed differential expression under waterlogging compared to the control condition. Among these DEGs, the number of upregulated genes with higher expression levels in CT compared with that in CK was 3455 (50.53%), while that of the downregulated ones with lower expression levels in CT was 3382 (49.47%) (electronic supplementary material, table S2). Of the transcripts with varied expressions, 233 transcripts were expressed only in the CK library, 225 transcripts only in the CT library (electronic supplementary material, table S3) and 6379 transcripts were expressed in both libraries, which indicated that waterlogging and low-oxygen condition could activate or repress quite a number of unique transcripts.

### 3.5. Functional classification of differentially expressed genes

Genes with altered expression responses spanned a wide variety of regulatory and metabolic processes. To further evaluate the response to waterlogging, GO enrichment analysis was performed to categorize the upregulated and downregulated DEGs with the whole transcriptome as the background.

**Figure 3.**
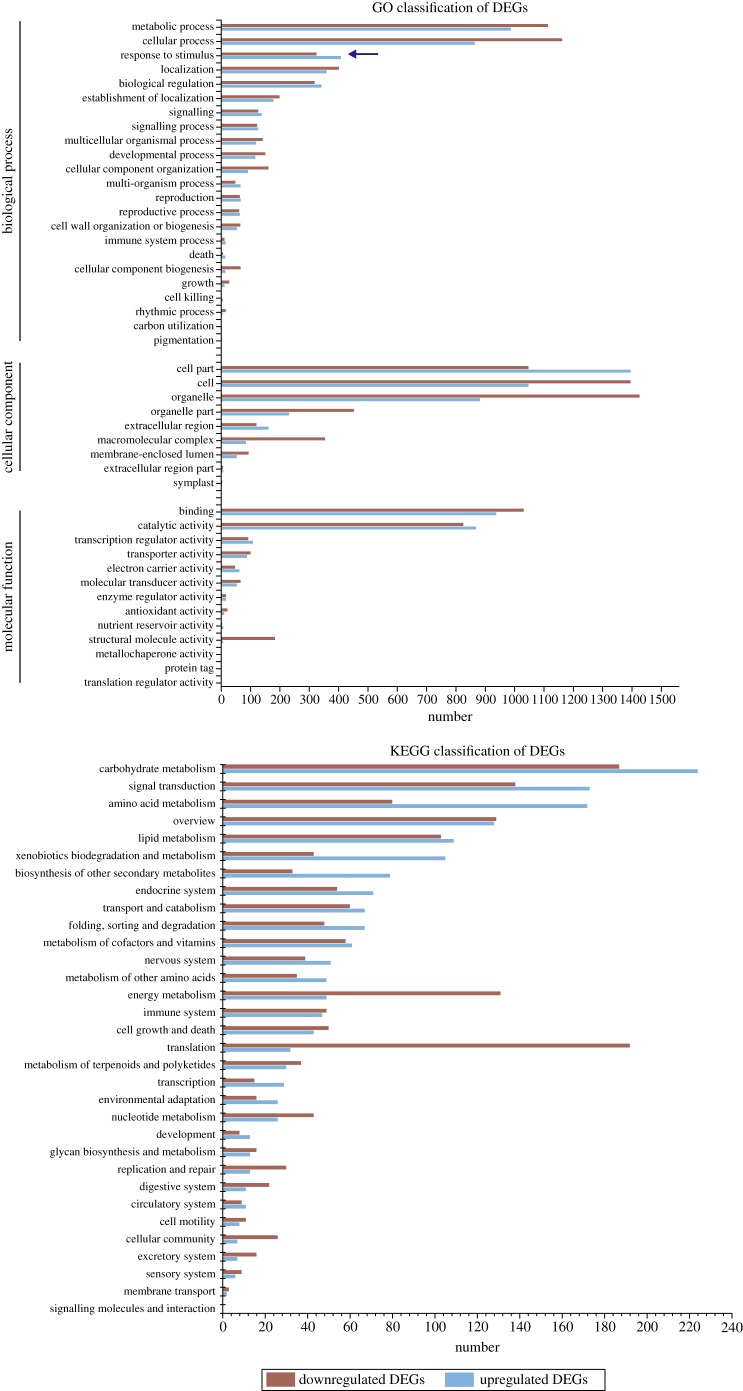
Comparison between upregulated and downregulated genes based on GO function categories and KEGG pathway analysis.

The analysis indicated that 3115 DEGs (45.6%) could be appointed to GO annotation categories; of these, 1456 were upregulated and 1659 were downregulated DEGs. All these categorized genes were classified into 46 functional groups, including 23 groups in BP, 14 groups in MF and 9 in CC. In the MF category, the top three enriched terms were ‘binding' (1970 transcripts of 6387), ‘catalytic activity' (1695 transcripts of 6387) and ‘structural molecule activity' (191 transcripts of 6387). In the CC category, ‘cell’ (2444 of 6387), ‘cell part' (2444 of 6387) and ‘organelle' (1625 of 6387) were the three dominant enriched terms. In the category of BP, ‘metabolic process' (2103 transcripts), ‘cellular process' (2029 transcripts) and ‘biological regulation' (662 transcripts) were the mostly highly enriched (figure 3). Totally, GO enrichment analysis of the DEGs indicated that the most highly enriched functional categories were ‘cell', ‘cell part', ‘metabolic process', ‘cellular process’, ‘binding', ‘catalytic activity' and ‘organelle'. The DEGs related to organelle, catalytic activity, binding, cellular process, metabolic process, cell, cell part were abundant in the DEGs. It was also found that approximately 10.8% (735 transcripts of 6387) of the DEGs were those responding to stimulus (figure 3).

Comparison between these categories indicated that downregulated genes were more than the upregulated ones under the waterlogging condition in most categories (figure 3). However, there was also the phenomenon that the upregulated genes in response to waterlogging were more than the downregulated in some categories, such as the categories of ‘electron carrier activity', ‘transcription regulator activity', ‘signaling', ‘biological regulation' and ‘response to stimulus'. As shown in figure 3, the top three largest groups of upregulated genes were ‘cell part', ‘cell' and ‘metabolic process’, while ‘cell part', ‘cell' and ‘cellular process’ were the top three largest groups of downregulated genes.

To characterize the functional consequences of gene expression changes associated with waterlogging stress, a pathway analysis of the DEGs based on the KEGG database was performed. Nearly 20.7% (1416) DEGs were mapped to pathways, whereas the rest were unassigned. The top-seven enriched pathways by DEGs were ‘Carbohydrate metabolism', ‘Translation', ‘Signal transduction’, ‘Overview', ‘Amino acid metabolism', ‘Energy metabolism' and ‘Lipid metabolism’ (figure 3). Obviously, there were more downregulated genes than upregulated ones in the ‘Translation' and ‘Energy metabolism’ pathways, and similarly, there were more upregulated genes than downregulated ones in the ‘Biosynthesis of other secondary metabolites' and ‘Amino acid metabolism' pathways.

### 3.6. Chlorophyll metabolism and photosynthetic capabilities of grapevine in response to waterlogging stress

**Figure 4.**
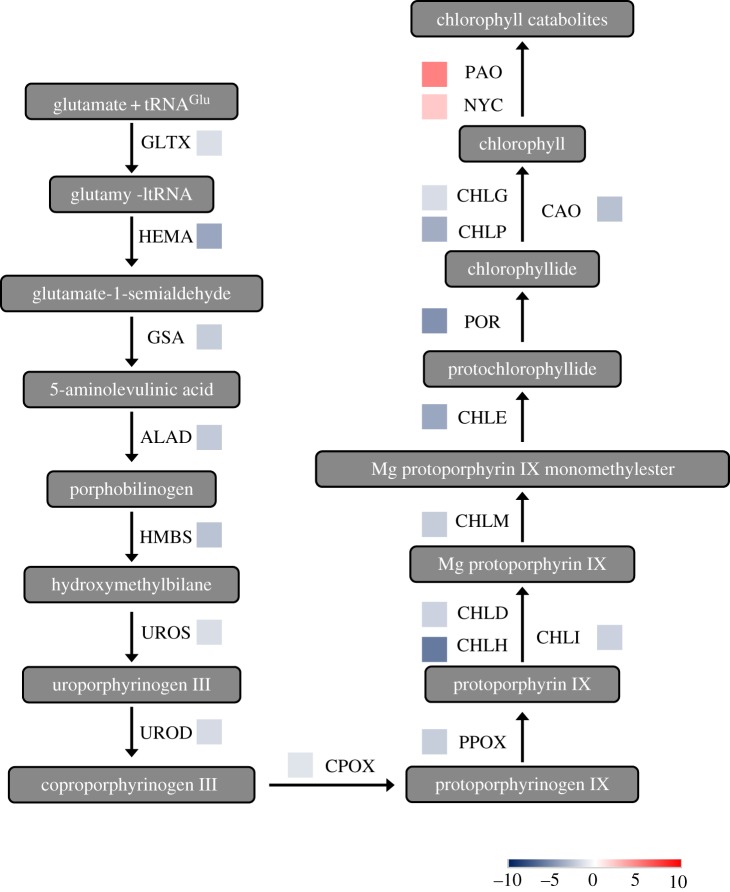
Schematic diagram of chlorophyll metabolism proposed during plant waterlogging stress. tRNA^Glu^, glutamyl-transfer RNA; glutamyl-tRNA synthetase, GLTX; glutamyl-tRNA reductase, HEMA; glutamate-1-semialdehyde 2,1-aminomutase, GSA; 5-aminolevulinic acid dehydratase, ALAD; hydroxymethylbilane synthase, HMBS; uroporphyrinogen-III synthase, UROS; uroporphyrinogen decarboxylase, UROD; coproporphyrinogen III oxidase, CPOX; protoporphyrinogen oxidase, PPOX; magnesium-chelatase subunit H, CHLH; magnesium-chelatase subunit D, CHLD; magnesium-chelatase subunit I, CHLI; magnesium-protoporphyrin O-methyltransferase, CHLM; magnesium-protoporphyrin IX monomethylester oxidative cyclase, CHLE; protochlorophyllide oxidoreductase, POR; chlorophyll synthase, CHLG; chlorophyllide a oxygenase, CAO; geranylgeranyl reductase, CHLP; chlorophyllide b reductase, NYC; pheophorbide a oxygenase, PAO. The colour of the boxes represents the fold-change value of DEG. The number of boxes represents the number of DEGs. Data can be found in electronic supplementary material, table S4.

**Figure 5.**
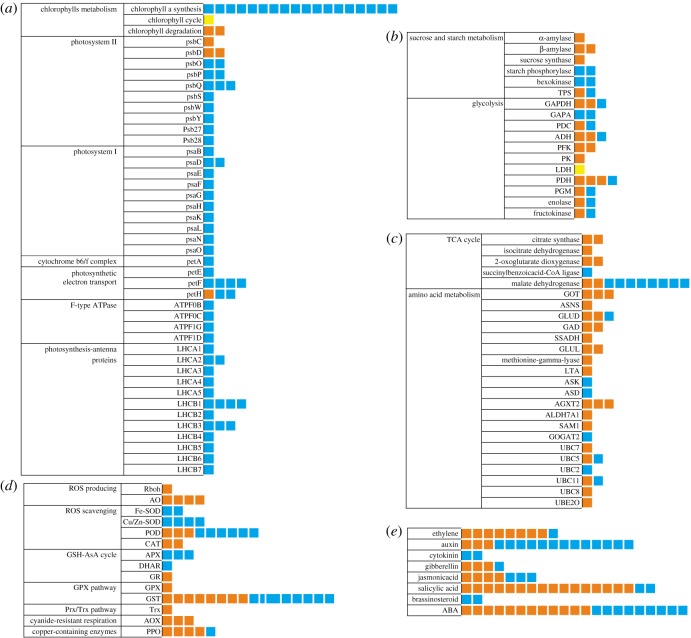
Overview of expression changes in various metabolic pathways induced by waterlogging stress. The upregulated DEGs are marked in red. The downregulated DEGs are marked in blue. Genes that hardly changed are coloured yellow.

In this study, quite a number of genes implicated in photosynthesis, the expressions of which were changed significantly under waterlogging conditions, were identified. The chlorophyll metabolic pathway mainly comprised three phases: (I) chlorophyll a synthesis from glutamate, (II) interconversion of chlorophyll a and chlorophyll b (chlorophyll cycle), and (III) chlorophyll degradation pathway. According to the grapevine transcriptome data, 20 transcripts involved in chlorophyll metabolism showed significant differences in response to low-oxygen stress compared with control, including 18 downregulated DEGs and two upregulated DEGs. Meanwhile, 20 transcripts (glutamyl-tRNA synthetase, GLTX; glutamyl-tRNA reductase, HEMA; glutamate-1-semialdehyde 2,1-aminomutase, GSA; 5-aminolevulinic acid dehydratase, ALAD; hydroxymethylbilane synthase, HMBS; uroporphyrinogen-III synthase, UROS; uroporphyrinogen decarboxylase, UROD; coproporphyrinogen III oxidase, CPOX; protoporphyrinogen oxidase, PPOX; magnesium-chelatase subunit D, CHLD; magnesium-chelatase subunit H, CHLH; magnesium-chelatase subunit I, CHLI; magnesium-protoporphyrin O-methyltransferase, CHLM; magnesium-protoporphyrin IX monomethylester oxidative cyclase, CHLE; protochlorophyllide oxidoreductase, POR; chlorophyll synthase, CHLG; geranylgeranyl reductase, CHLP) in chlorophyll a synthesis and one single transcript (chlorophyllide a oxygenase, CAO) in the chlorophyll cycle were significantly decreased, and a single transcript (chlorophyllide b reductase, NYC; pheophorbide a oxygenase, PAO) in the chlorophyll degradation pathway was significantly increased (figures 4 and 5*a*; electronic supplementary material, table S4). The results indicated that the exogenous low-oxygen condition from waterlogging stress could inhibit chlorophyll synthesis and induce the chlorophyll degradation that caused a decrease in chlorophyll content.

From grapevine transcriptome data, 57 photosynthesis-related genes sensitive to waterlogging stress were identified, which were involved in PSII (15 transcripts), PSI (11), cytochrome b6/f complex (1), ATP synthase (4), photosynthetic electron transport chain (8) and photosynthesis–antenna proteins (18) (figure 5*a*; electronic supplementary material, table S4 and figure S1). Among the 15 DEGs in photosystem II, two psbOs and psbPs, three psbQs and single genes (psbS, psbW, psbY, psb27 and psb28) were significantly decreased, while psbC and two psbD were found to be increased compared with those in control (figure 5*a*; electronic supplementary material, table S4). The expression levels of 11 genes related to PSI and one transcript involved in cytochrome b6-f complex (one petA) showed significant reduction with the control group (figure 5*a*; electronic supplementary material, table S4). Four ATP synthase genes were also significantly downregulated (figure 5*a*; electronic supplementary material, table S4). Of the genes involved in the photosynthetic electron transport chain, the expressions of one petE gene, two petH genes and four petF genes were found to be inhibited, while one petH (VIT_10s0003g04880.t01) showed significant increase compared with those in the control group (figure 5*a*; electronic supplementary material, table S4). All 18 photosynthesis–antenna proteins had also greatly decreased expression.

### 3.7. Differential regulation of genes associated with anaerobic fermentation and glycolysis

**Figure 6.**
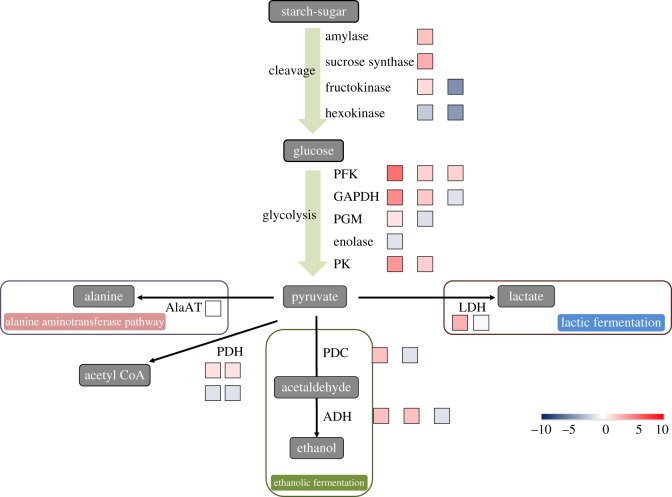
Schematic diagram of starch metabolism, glycolysis and fermentation proposed during plant waterlogging stress. AlaAT, alanine aminotransferase; TPS, trehalose 6-phosphate synthase; GAPDH, glyceraldehyde 3-phosphate dehydrogenase; GAPA, glyceraldehyde-3-phosphate dehydrogenase (NADP^+^); PDC, pyruvate decarboxylase; ADH, alcohol dehydrogenase; PFK, 6-phosphofructokinase; PK, pyruvate kinase; LDH, lactic dehydrogenase; PDH, pyruvate dehydrogenase; PGM, phosphoglucomutase. The colour of the boxes represents the fold-change value of DEG. The number of boxes represents the number of DEGs. Data can be found in electronic supplementary material, table S5.

The changes in carbon metabolism-related genes were apparent in leaves. Starch and sucrose metabolism were the top-five enriched pathways by DEGs in CT leaves, compared with CK (figure 6). Therefore, starch and sucrose metabolisms were also found to be affected by waterlogging. There were 60 transcripts annotated as involved in this pathway. We found that waterlogging promoted the expression of the genes involved in sugar and starch cleavage: one α-amylase transcript (VIT_03s0063g00400.t01), two β-amylase transcripts (VIT_05s0077g00280.t01 and VIT_02s0012g00170.t01) and single sucrose synthase transcripts (VIT_04s0044g00020.t01) were upregulated. By contrast, waterlogging inhibited the accumulation of mRNAs encoding for enzymes controlling starch synthesis: hexokinase (VIT_06s0061g00040.t01 and VIT_18s0001g14230.t01). There were two transcripts annotated as encoding trehalose 6-phosphate synthase (TPS): VIT_12s0028g01670.t01 and VIT_01s0026g00280.t01 with 1.78- and 1.93-fold increased expression, respectively, involved in starch and sucrose metabolism (figures 6 and 5*b*; electronic supplementary material, table S5).

Perturbation in glycolysis is considered to be the basic characteristic of plant adaption to an aerobic stress [34]. There were 47 transcripts (24 transcripts were upregulated and 23 transcripts were downregulated) annotated as encoding enzymes involved in the glycolysis/gluconeogenesis pathway between treated and control leaves. Among these transcripts, the expression of the genes associated with the glycolysis pathway, which produces two ATP and two pyruvate molecules per unit of hexose while concomitantly reducing NAD^+^ to NADH, was upregulated by waterlogging stress. Three transcripts were annotated as encoding *glyceraldehyde 3-phosphate dehydrogenase* (GAPDH): VIT_19s0085g00600.t01, VIT_01s0010g02460.t01 and VIT_17s0000g10430.t01; and two transcripts were annotated as encoding *glyceraldehyde-3-phosphate dehydrogenase (NADP+) (phosphorylating)* (GAPA): VIT_14s0068g00680.t01 and VIT_18s0122g00960.t01. However, one GAPDH (VIT_17s0000g10430.t01) (from 560.17 to 1269.08 RPKM) and two GAPAs (from 346.55 to 1232.57 RPKM, from 263.18 to 3259.16 RPKM, respectively) were the downregulated transcripts and have the higher abundance than the upregulated transcripts, indicating that an additional GAPDH isoform may be inhibited in hypoxic leaves. In addition, phosphofructokinase (PFK) (three transcripts) and pyruvate kinase (PK) (one transcript) were upregulated (figures 6 and 5*b*; electronic supplementary material, table S5).

Waterlogging stress induces anaerobic metabolism in the leaves of waterlogged seedlings. The accumulation of alcohol is less toxic than that of lactic acid, and thus the timing of the shift to lactic from alcoholic fermentation is considered to represent an important indicator of the ability of a plant to survive hypoxia without suffering extensive cellular damage. Three critical enzymes are involved in the process of alcoholic fermentation: lactate dehydrogenase (LDH), which first catalyses the conversion of lactate to pyruvate; pyruvate decarboxylase (PDC), which then converts pyruvate to acetaldehyde; and alcohol dehydrogenase (ADH), which further metabolizes acetaldehyde to ethanol. In our results, two PDC transcripts (one upregulated and one downregulated) and three ADH transcripts (two upregulated and one downregulated) were identified. The upregulated PDC and ADH were expressed at high abundance (for example, PDC: VIT_10s0003g00990.t01, from 168.02 to 30.98 RPKM; ADH: VIT_18s0001g15410.t01, from 3136.46 to 74.41 RPKM), while the others had low transcript abundances. As expected, an increase in the expression of fermentative genes (PDC and ADH) was evident. However, LDH (two transcripts) show no change in the expression level and have a low abundance (e.g. VIT_15s0048g00640.t01, from 0.34 to 0.04 RPKM) (figures 6 and 5*b*; electronic supplementary material, table S5). By activating alcoholic fermentation, energy was produced in waterlogged leaves. This was consistent with findings for grey poplar [9].

### 3.8. Differential regulation of genes associated with amino acid metabolism

**Figure 7.**
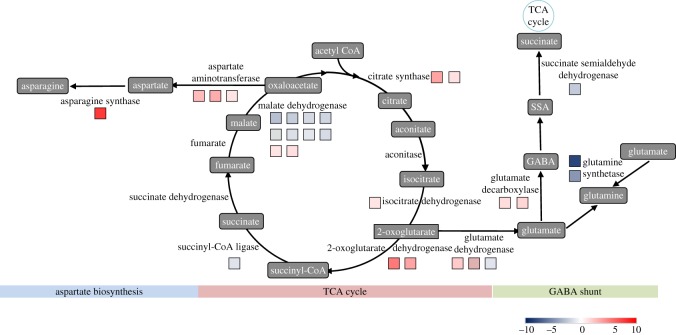
Schematic diagram of tricarboxylic acid (TCA) cycle, caminobutyric acid (GABA) shunt and amino acid metabolism proposed during plant waterlogging stress. The colour of the boxes represents the fold-change value of DEG. The number of boxes represents the number of DEGs. Data can be found in electronic supplementary material, table S6.

In theory, breakdown of carbohydrates is the main pathway of carbon flux and energy supply. Actually, intermediates from lipid and protein degradation also act as important carbon sources entering glycolysis under waterlogging. Many genes encoding proteins or enzymes involved in degradation of lipids and proteins were identified in our transcriptome library, which supports the hypothesis mentioned above. It is interesting that about 10% of the genes induced by waterlogging were related to protein metabolism, including synthesis and degradation. The waterlogging stress induced the upregulation of genes encoding aldehyde dehydrogenase family 7 member A1 (ALDH7A1) (VIT_11s0016g00900.t01, from 113.29 to 17.99 RPKM). The gene encoding S-adenosylmethionine decarboxylase (VIT_08s0007g05000.t01, from 132.56 to 16.28 RPKM) was also upregulated in the leaves of waterlogged seedlings (figures 7 and 5*c*; electronic supplementary material, table S6). This protein is a key enzyme involved in the biosynthesis of the polyamines spermidine and spermine, and is also known to influence the rate of ethylene (ETH) biosynthesis [35]. These data indicate that response to oxygen deprivation occurs at the whole-plant level.

Furthermore, many genes related to protein degradation were upregulated. The expression of alanine-glyoxylate transaminase (AGXT) (three transcripts), an enzyme responsible for conversion of alanine to pyruvate, was found upregulated. Eleven ubiquitin-conjugating enzyme transcripts (seven upregulated and four downregulated) (figure 7 and 5*c*; electronic supplementary material, table S6) that perform the second step in the ubiquitination reaction that targets a protein for degradation were identified.

Surprisingly and importantly, the expression of the alanine aminotransferase (AlaAT) transcript, which is involved in amino acid metabolism, was not changed in leaf cells of grape seedlings under waterlogging conditions. AlaAT catalyses reversible transfer of an amino group from alanine to 2-oxoglutarate to form pyruvate and glutamate. In other words, the accumulation of alanine not responding to the waterlogging stress among grapevine leaves.

The gene encoding ferredoxin-dependent glutamate synthase (GS) (VIT_08s0007g05260.t01, 47.85 to 17.21 RPKM) was also upregulated in response to waterlogging. GS is involved in the GS/GOGAT cycle, which is used to store carbon and nitrogen, and is responsible for regenerating glutamate [36]. Glutamate has been shown to be a critical product for regulation of cytoplasmic pH. It is catalysed by glutamate decarboxylase (GAD) (two upregulated transcripts, VIT_17s0000g00920.t01 and VIT_01s0011g06600.t01) (figures 7 and 5*c*; electronic supplementary material, table S6), consuming a proton and synthesizing GABA (γ-aminobutyric acid) [37,38]. GABA can be converted to succinate and enters carbon metabolism. This metabolic pathway involves GABA-transaminase and SSADH (succinate-semialdehyde dehydrogenase), and this route for glutamate carbon to enter the tricarboxylic acid cycle (TCA cycle) is called the GABA shunt. Alanine is synthesized from pyruvate in this process, which suggests the GABA shunt is responsible for accumulation of alanine under waterlogging conditions (figures 7 and 5*c*; electronic supplementary material, table S6).

Considering the information above, it is possible that alanine can be accumulated from protein degradation and the GABA shunt, and that there is a cycle between pyruvate and alanine. In this study, a gene encoding aspartate aminotransferase (three transcripts) was shown to be induced under waterlogging, which can generate glutamate and oxalacetic acid (OAA) from aspartate and 2-oxoglutarate. OAA can be converted to malate, which has a critical role in regulation of cytoplasmic pH, catalysed by malic enzyme, which was also upregulated in this study.

### 3.9. Differential regulation of genes associated with reactive oxygen species generation/scavenging

From the transcriptome data, many genes encoding these enzymes involved in the ROS generation and scavenging system were significantly changed by the waterlogging stress. The components of the antioxidant defence system are enzymatic and non-enzymatic antioxidants. Enzymatic antioxidants include SOD, CAT, APX, MDHAR, DHAR and GR and non-enzymatic antioxidants are GSH, AA (both water soluble), carotenoids and tocopherols (lipid soluble) [39].

Sixty-nine transcripts with differentially expressed profiles were identified as encoding enzymes in the ROS scavenging system. They were categorized into the Cu–Zn superoxide dismutase (Cu/Zn-SOD, three transcripts), peroxidase (POD, 9), catalase (CAT, 2), glutathione-ascorbate (GSH-AsA) cycle (5), glutathione peroxidase (GPX, 1), glutathione S-transferase (GST, 16) and the thioredoxin (Trx) pathways (1), alternative oxidase (AOX, 3) and polyphenol oxidase (PPO, 5) (figure 5*d*; electronic supplementary material, table S7).

**Figure 8.**
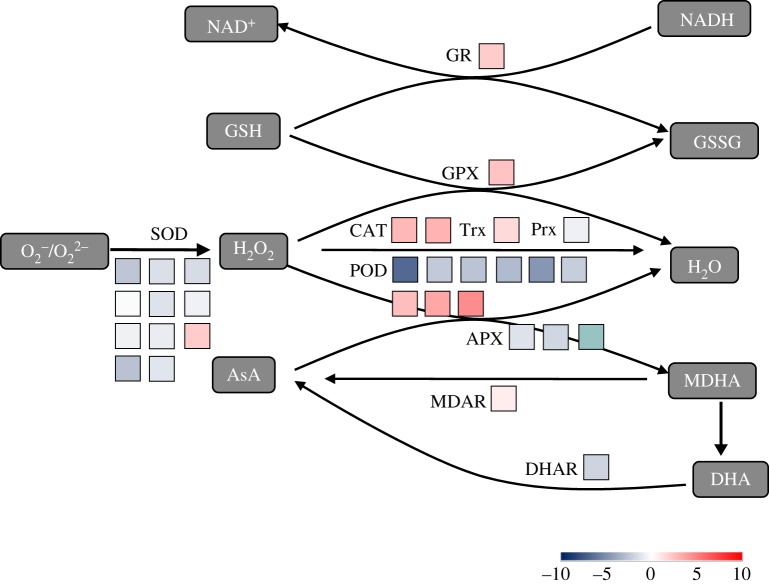
Schematic diagram of ROS generation/scavenging proposed during plant waterlogging stress. SOD, superoxide dismutase; CAT, catalase; APX, ascorbate peroxidases; MDAR, monodehydroascorbate reductase; DHAR, dehydroascorbate reductase; GR, glutathione reductase; GPX, glutathione peroxidases; ${{\text{O}}_{2}}^{*-},$ superoxide radicals; H_2_O_2_, hydrogen peroxide; ${\;}^{1/2}{\text{O}}_{2}$, singlet oxygen; GSSG, oxidized glutathione; MDHA, monodehydroascorbate; AsA, ascorbic acid; DHA, dehydroascorbate; GSH, glutathione. The box colour represents the fold-change value of DEG. The number of boxes represents the number of DEGs. Data can be found in electronic supplementary material, table S7.

SODs provide the first line of defence mechanism against highly toxic superoxide radicals. SOD catalyses the removal of ${{\text{O}}_{2}}^{-}$ by dismutating it into O_2_ and H_2_O_2_ (figure 8). SODs are classified by metal cofactors into three types: copper–zinc (Cu/Zn-SOD), iron (Fe-SOD) and manganese (Mn-SOD), which are localized in different cellular compartments. In our results, two Fe-SODs, two Mn-SODs and one copper chaperone for superoxide dismutase (CCS) showed no changes of expression levels (|log_2_FC| < 1), while three Cu/Zn-SODs were remarkably downregulated (|log_2_FC| > 2). One of the downregulated Cu-Zn SOD genes, VIT_06s0061g00750.t01 (from 137.61 to 764.22 RPKM), had high expression abundance, and the other two downregulated Fe-SOD genes had low transcript abundance (e.g. VIT_10s0042g00100.t01, from 3.81 to 24.85 RPKM) (electronic supplementary material, table S7). The results supported the fact that the function of Fe-SOD can be substituted by Cu/Zn-SOD.

CAT and POD took part in the metabolism reducing H_2_O_2_ directly to water and oxygen (oxide donors) (figure 8). In this study, two CATs and three PODs were upregulated by waterlogging stress, while six PODs were downregulated, suggesting the importance of CATs and not PODs in scavenging ROS under waterlogging stress (figure 5*d*; electronic supplementary material, table S7). Two upregulated CAT genes (VIT_00s0698g00010.t01, from 1993.52 to 296.15 RPKM; VIT_04s0044g00020.t01, from 3066.80 to 394.80 RPKM) had very high abundance (electronic supplementary material, table S7). Among the nine identified POD genes, three upregulated genes, VIT_07s0129g00360.t01 (from 23.51 to 4.02 RPKM), VIT_18s0072g00160.t01 (from 103.44 to 9.69) and VIT_12s0055g01010.t01 (from 14.56 to 0.67 RPKM), had low abundance and other downregulated genes were expressed at quite high levels in CK, such as VIT_10s0116g01780.t01 (from 180.76 to 810.82) (electronic supplementary material, table S7).

Moreover, the non-enzymatic antioxidant defence system including ascorbate–glutathione (AsA-GSH) cycle and the GPX pathway was identified, which also played essential roles in the defence against ROS and H_2_O_2_ induced by waterlogging stress (figure 8). Totally, four AsA-GSH cycle genes (including APX, GR and DHAR) were identified, including one upregulated gene and three downregulated genes (figures 8 and 5*d*; electronic supplementary material, table S7). Sixteen glutathione S-transferase transcripts (GSTs, eight upregulated, eight downregulated) were identified in the GPX pathway (figure 5*d*; electronic supplementary material, table S7). Two transcripts, VIT_12s0028g00920.t01 (|log_2_FC|=6.50) and VIT_07s0005g04880.t01 (|log_2_FC|=4.34), had significantly induced expression in the waterlogging-treated sample (electronic supplementary material, table S7). Furthermore, there was one upregulated Trx in the Trx pathway (figures 8 and 5*d*; electronic supplementary material, table S7). In addition, all three alternative oxidases (AOs) were significantly upregulated in the cyanide-resistant respiration. Four of five polyphenol oxidase (except for one downregulated PPO) transcripts were ubiquitous copper-containing enzymes and also were significantly upregulated compared with control (figure 5*d*; electronic supplementary material, table S7).

### 3.10. Differential regulation of genes related to hormone metabolism

Plant hormones play pivotal roles in plant stress signalling and function as central integrators that link and reprogramme the complex developmental and stress adaptive signalling cascades. Multiple hormones are involved in waterlogging stress, such as ETH, ABA, salicylic acid and jasmonic acid (JA). In this transcriptome result, many genes involved in the auxin (IAA), ETH (ETH), JA (JA), abscisic acid (ABA), brassinolide (BR) and gibberellin (GA) synthesis and signal transduction pathways were detected (figure 5*e*; electronic supplementary material, table S8). These pathways play well-established roles in plant defence against stress.

**Figure 9.**
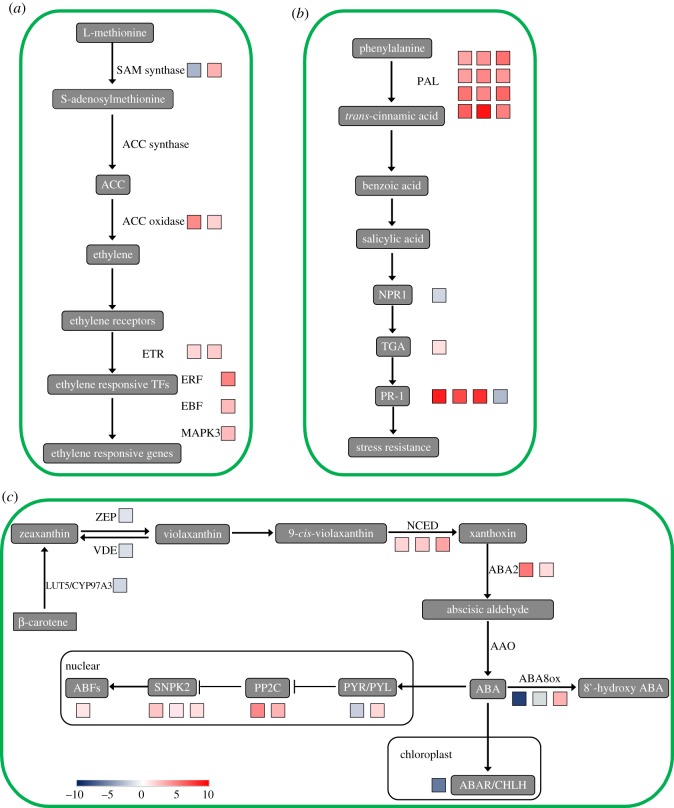
Schematic diagram of ethylene synthesis (*a*), salicylic acid synthesis (*b*), ABA synthesis (*c*) and signal transduction proposed during plant waterlogging stress. (*a*) ACC oxidase, 1-aminocyclopropane-1-carboxylic acid oxidase 2; SAM synthase, S-adenosylmethionine synthetase; ETR, ethylene receptor; EBF, EIN3-binding F-box protein; MAPK, mitogen-activated protein kinase; ERF, ethylene-responsive transcription factor 1. (*b*) PAL, phenylalanine ammonia-lyase; NPR1, regulatory protein NPR1; TGA, transcription factor TGA; PR1, pathogenesis-related protein 1. (*c*) LUT5/CYP97A3, β-ring hydroxylase; ZEP, zeaxanthin epoxidase; VDE, violaxanthin de-epoxidase; NCED, 9-*cis*-epoxycarotenoid dioxygenase; ABA8ox, abscisic acid 8′-hydroxylase; ABA2, xanthoxin dehydrogenase; PYL, abscisic acid receptor PYR/PYL family; PP2C, protein phosphatase 2C; SNRK2, serine/threonine-protein kinase SRK2; ABF, ABA responsive element binding factor; CHLH, magnesium chelatase subunit H. The colour of the boxes represents the fold-change value of DEG. The number of boxes represents the number of DEGs. Data can be found in electronic supplementary material, table S8.

ETH is involved in responses to hypoxia and is considered to be a contributor to adventitious root production and aerenchyma formation [40]. Our analysis demonstrated the differential regulation of DEGs encoding for proteins functioning in ETH synthesis and response. The expression of the key gene controlling ETH synthesis, 1-aminocyclopropane-1-carboxylate (ACC) oxidase (ACO) (VIT_12s0059g01380.t01) and S-adenosylmethionine synthetase (VIT_08s0007g05000.t01) was upregulated under waterlogging conditions. In addition, we found waterlogging-induced accumulation of transcripts encoding for ETH receptor (ETR) (VIT_07s0005g00850.t01, VIT_05s0049g00090.t01). Interestingly, one gene encoding for ERF (ETH responsive factors) (VIT_05s0049g00510.t01) transcription factors, being crucial to the ETH signalling pathway, was upregulated by waterlogging (figure 9*a*; electronic supplementary material, table S8). This indicates that ETH could play an important role in the waterlogging response of grape.

Some auxin-induced genes were upregulated under waterlogging stress, such as GH3 (auxin-conjugating enzyme) (three transcripts), while some genes involved in auxin metabolism (synthesis as well as degradation) and auxin signal transduction were downregulated, such as ARF (auxin response factor, one transcripts) and IAA (auxin-responsive protein, eight transcripts) (electronic supplementary material, table S8). The auxin synthesis-related gene aldehyde dehydrogenase (ALDH) was upregulated in response to waterlogging stress (electronic supplementary material, table S6). These results suggest that auxin is involved in grapevine waterlogging responses, and may act as an important signal-mediating response to waterlogging.

Cytokinin levels decreased in the xylem sap of plants subjected to waterlogging, and these factors have been posited to have a role in signalling root waterlogging to the aerial part of the plant [41,42]. However, the involvement of these hormones was not well supported by the DGE analyses of gene expression in grapevine leaf. For example, two cytokinin receptors (VIT_12s0057g00690.t01 and VIT_01s0011g06190.t01) were downregulated. These results are more in keeping with the recent work that has suggested that these plant hormones do not play major roles in the hypoxic response [1].

JA and salicylic acid (SA) have been shown to possess crucial functions in mediating or orchestrating stress responses in plants. Some genes related to JA pathways were also identified, including genes involved in jasmonate signalling and biosynthesis, two upregulated JAZ (jasmonate-ZIM domain) protein transcripts, four LOX (lipoxygenase) transcripts (three upregulated and one downregulated) and one downregulated AOC (allene oxide synthase) transcript (electronic supplementary material, table S8). Furthermore, a large number of genes involved in SA synthesis and those mediated by SA signal transduction were significantly upregulated under waterlogging stress (figure 9*b*; electronic supplementary material, table S8). For example, 12 upregulated genes (phenylalanine ammonia-lyase, a key enzyme in the SA biosynthesis pathway involved in the response to diverse stresses) were identified. We also found that two transcripts related to brassinosteroid synthesis were significantly downregulated under waterlogging stress: SMT1 (sterol methyltransferase, VIT_13s0064g00440.t01) and BAK1 (BRASSINOSTEROID INSENSITIVE 1-associated receptor kinase 1, VIT_18s0164g00070.t01) (electronic supplementary material, table S8).

Waterlogging can induce substantial ABA accumulation in leaves, which is mainly responsible for the severe stomatal closure following waterlogging in plants. Our analysis also found that ABA metabolic and signal transduction pathways downregulated the expression of carotenoid beta-ring hydroxylase (LUT5/CYP97A3) (VIT_04s0023g00080.t01), zeaxanthin epoxidase (ZEP) (VIT_07s0031g00620.t01), violaxanthin de-epoxidase (VDE) (VIT_04s0043g01010.t01) and magnesium chelatase subunit H (CHLH) (VIT_08s0007g08540.t01), and upregulated the expression of 9-*cis*-epoxycarotenoid dioxygenase (NCED) (three transcripts), protein phosphatase 2C (two transcripts) under waterlogging stress. In addition, three abscisic acid 8'-hydroxylase genes (ABA8ox) (two downregulated and one upregulated), which are involved in abscisic acid inactivation [43], were identified in the leaf as having decreased expression (figure 9*c*; electronic supplementary material, table S8),

### 3.11. Regulation of transcripts encoding non-symbiotic haemoglobins

A special class of hypoxia-responsive proteins that have always attracted special attention because of their oxygen-binding properties are the haemoglobins. Haemoglobins are haeme-containing proteins found in most organisms including animals, bacteria and plants. Haemoglobins are one of many different strategies that plants have evolved to overcome stress conditions and survive. There are three different types of haemoglobins in plants: symbiotic, non-symbiotic and truncated haemoglobins. The non-symbiotic haemoglobins (nsHb) are divided into: class 1 haemoglobins, which have a very high affinity for oxygen; and class 2 haemoglobins, which have lower affinity for oxygen [44]. The nsHb are expressed under hypoxia, osmotic stress, nutrient deprivation, cold stress, rhizobial infection, nitric oxide exposure and fungal infection. In the study, we identified two upregulated unigenes (VIT_03s0063g01970.t01, VIT_03s0063g01960.t01) annotated as encoding class 2 nsHb (electronic supplementary material, table S9), respectively, increased 2.27-fold and 4.41-fold in CT leaves. This may facilitate grapevine waterlogging tolerance. Additionally, mRNAs related to haeme (i.e. nsHb) binding were highly upregulated in the CT leaves.

Haemoglobins also react with NO produced under different stress conditions. Class 2 nsHbs are involved in a metabolic pathway involving NO. Those haemoglobins provide an alternative type of respiration to mitochondrial electron transport under limiting oxygen concentrations. Class 2 nsHb in hypoxic plants act as part of a soluble, terminal, NO dioxygenase system, yielding nitrate from the reaction of oxyhaemoglobin with NO [45]. The overall reaction sequence, referred to as the nsHb/NO cycle, consumes NADH and maintains ATP levels via an as yet unknown mechanism. Therefore, we evaluated the regulation of the genes involved in nitrate metabolism and found that a set of genes showed no change in response to waterlogging in grapevine leaves. These genes encode nitrate reductase (NR) (VIT_18s0001g03910.t01, from 130.52 to 92.36 RPKM), a key enzyme responsible for conversion of nitrate $\left(\text{N}{{\text{O}}_{3}}^{-}\right)$ to nitrite $\left(\text{N}{{\text{O}}_{2}}^{-}\right)$ and nitric oxide (NO); and nitrite reductase (NiR) (VIT_03s0063g00370.t01, from 56.31 to 65.89 RPKM), an enzyme responsible for reduction of $\text{N}{{\text{O}}_{2}}^{-}$ to ammonium and conversion of $\text{N}{{\text{O}}_{2}}^{-}$ to NO, and $\text{N}{{\text{O}}_{3}}^{-}$ transporter (electronic supplementary material, table S9). These results suggest that the regulation of $\text{N}{{\text{O}}_{3}}^{-}$ metabolism and the modulation of endogenous NO levels might be important for waterlogging acclimation in grapevine. Furthermore, we also identified 3 haeme-binding protein transcripts (two upregulated and one downregulated) (electronic supplementary material, table S9). Thus, more attention needs to be paid to the role of nsHb in grapevine waterlogging tolerance.

### 3.12. Damage severity prediction candidate gene selection and verification of differentially regulated genes by real-time polymerase chain reaction

**Figure 10.**
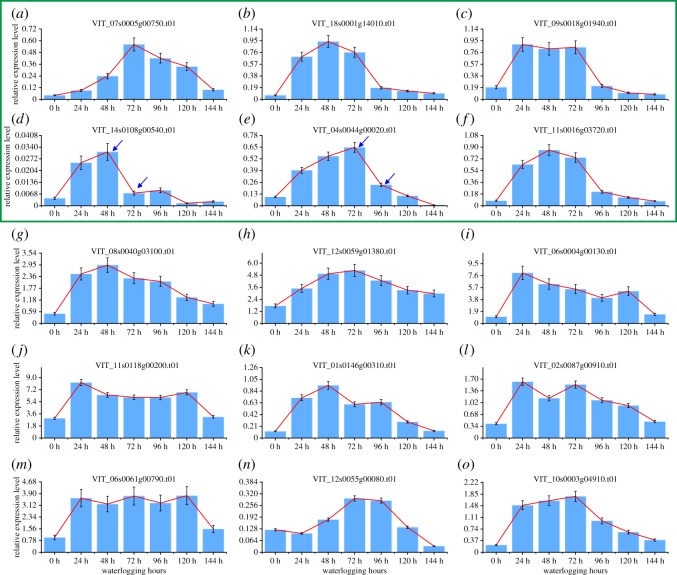
(*a*–*o*) Expression patterns of the 15 candidate upregulated genes under waterlogging stress.

**Figure 11.**
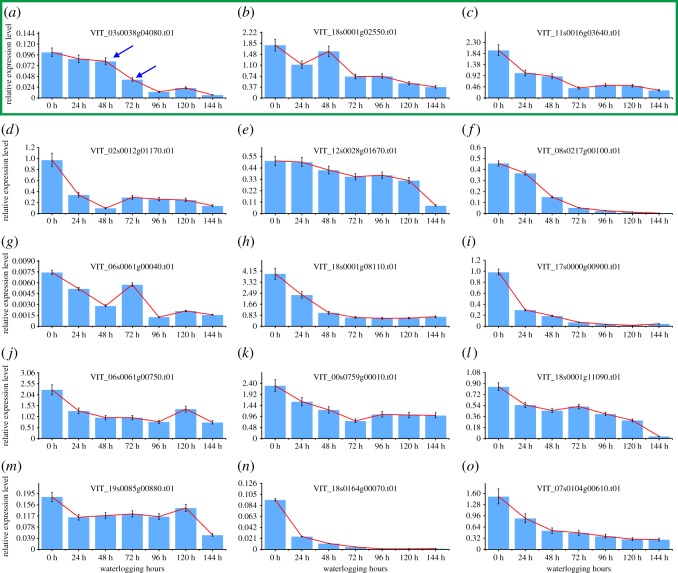
(*a*–*o*) Expression patterns of the 15 candidate downregulated genes under waterlogging stress.

To know if the gene expression signals could be used to show different damage severities caused by waterlogging stress, we select key genes that are sensitive to the variations of the status of grapevine under waterlogging stress. For this purpose, 30 highly DEGs (15 upregulated genes, 15 downregulated genes and one control *Actin*) were selected for analysis by qRT-PCR to determine their relative expression in response to waterlogging. The top differential expression genes were selected for analysis, so as to maximize the possibility of finding the optimal indicator genes, and also provide detailed spatio-temporal gene expression information under stress. These genes almost covered all the major functions identified in our transcriptome results, which include anaerobic fermentation and glycolysis, ROS generation/scavenging and plant hormone signalling, and some uncharacterized genes. The details of the primer sequences and gene functions are listed in electronic supplementary material, table S1. The expression patterns of these 30 genes in grapevine leaves, which were treated by 0, 24, 48, 72, 96, 120 and 144 h waterlogging, were analysed, and the results showed that under waterlogging stress, all the 30 tested genes were upregulated or downregulated consistent with the transcriptome results, which validated the accuracy and reproducibility of the transcriptome results. However, only nine of the 30 tested genes showed continual expression patterns under waterlogging stress, in which six genes showed a continually increasing expression trend (figure 10*a–f*) and three genes showed a continually decreasing expression trend (figure 11*a*–*c*) with the lasting waterlogging stress. Even if several downregulated genes (such as figure 11*d,f,h,i*) also showed continual expression patterns, these genes' expression decreased quickly, and we cannot observe the difference between the different treatment times. Thus these nine genes can be selected as candidates to further find out the expression signals corresponding to different damage severities caused by waterlogging stress.

### 3.13. Feasibility verification of using gene expression signals to predict the severity of damage caused to grapevine by waterlogging stress

**Figure 12.**
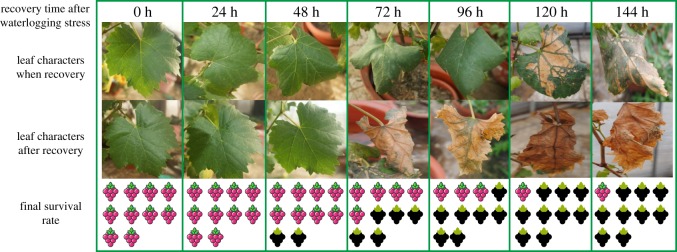
Grapevine growth statuses under waterlogging stress and recovery treatment. Grapevine plants were treated for 0, 24, 48, 72, 96, 120 and 144 h, respectively, and recovered by removing the water in the medium. Recovered plants were photographed 15 days after waterlogging stress was removed. 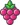 represents the surviving tree and 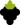 represents the tree that has died.

As the nine candidate genes have continual expression patterns under waterlogging stress, this means that every gene expression signal has only one occurrence during the whole response process under waterlogging stress. These features make them applicable to predict the severity of grapevine damage caused by waterlogging stress. To figure out the specific expression status of these candidate genes corresponding to different damage severities caused by waterlogging stress, we treated the grapevine with different time durations (0, 24, 48, 72, 96, 120 and 144 h) and then rescued them by removing the waterlogging stress. Then we observed the growth status of grapevine plants, and the results showed that, under waterlogging stress, grapevine leaves turned yellow and developed lesions with the lasting stress (figure 12) and eventually abscised. With regard to survival rate (figure 12), with less than 48 h of waterlogging treatment time, most grapevine plants (greater than 50%) can be rescued, and finally by removing the waterlogging stress, they can return to normal growth although some damaged leaves abscise a few days after recovery. However, with 72 h stress, the majority of grapevine plants (greater than 60%) eventually died finally after recovery, even though there is no evident death phenotype observed prior to applying recovery treatment. This critical time for waterlogging stress is 48 h. As the above-mentioned results, in this assay, the nine candidate genes showed increasing or decreasing expression trends with lasting waterlogging stress. Furthermore, we observed that under waterlogging stress, some of these genes appeared with some unique expression signals during the period of 48 h to 72 h of stress; for example, the expression level of gene VIT_14s0108g00540.t01 showed an increasing trend within 48 h of waterlogging stress, but a sharp decrease was observed at 72 h of waterlogging stress (figure 10*d*), and VIT_04s0044g00020.t01 showed an increasing trend within 72 h of waterlogging stress, but a sharp decrease was observed at 96 h of waterlogging stress (figure 10*e*); however, the expression level of VIT_07s0005g00750.t01 still was relatively high (figure 10*a*), and VIT_18s0001g14010.t01, VIT_09s0018g01940.t01 and VIT_11s0016g03720.t01 showed the same expression level during the period of 24 h to 72 h of waterlogging stress (figure 10*b*,*c*,*f*). In addition, the expression levels of VIT_03s0038g04080.t01 decreased slowly within 48 h of waterlogging stress, but decreased sharply at 72 h of waterlogging stress (figure 11*a*); however, the other genes (VIT_18s0001g02550.t01 and VIT_11s0016g03640.t01) decreased sharply with the time under waterlogging stress and soon the expression level was very low (figure 11*b*,*c*). As mentioned above, grapevine plants cannot be rescued by remedial measures over 72 h of waterlogging stress, which indicates that some genes showed similar expression features when the grapevine plants are damaged too much by waterlogging stress to be rescued by remedial measures; thus this expression feature of these three genes can be used as the marker signal to predict the severity of damage caused to grapevine by waterlogging stress. When genes VIT_14s0108g00540.t01 and VIT_04s0044g00020.t01 were detected with sharply increasing expression signals, and VIT_03s0038g04080.t01 was detected with sharply decreasing expression signals, it means that the grapevine plant has been damaged too much to be recovered by remedial measures.

## 4. Discussion

In the context of climate change, an increased frequency of waterlogging has been observed worldwide, which has an especially detrimental effect on crop production [10,46]; prolonged seasonal rainfall often causes severe yield losses of grapevine in central and southern China. To endure waterlogging stress, a series of rapid and profound molecular and metabolic responses will unavoidably be activated.

### 4.1. Global gene transcription changes in waterlogged grapevine leaves

In this paper, a global analysis of the transcriptome could facilitate the identification of systemic gene expression and regulatory mechanisms for the waterlogging tolerance of a plant. In the present study, a transcriptome profiling of the root was performed to identify genes that are differentially expressed in the early stage of waterlogging of grapevine. In this paper, transcriptomes of grapevine seedling leaves were sequenced using the Illumina platform. In total, about 29 million high-quality reads with a 5.99 Gb sequence coverage were obtained; there were 42 895 unigenes (greater than or equal to 200 bp) assembled and 69.87% were annotated to compare differential gene expression profiles of grapevine seedlings subjected to waterlogging, suggesting that the database selected is relatively complete.

Many of the genes are known to have responded to stimulus (figure 3). Similar root waterlogging experiments in poplar, cotton and *Artemisia annua* [1,9,47] have shown increased expression of glycolysis, fermentation and some catabolism pathways, and decreased expression of synthesis pathways, cell wall and secondary metabolism-associated genes. The gene transcription responses to waterlogging in grapevine resemble that in *Arabidopsis* subjected to O_2_ deprivation [7], indicating that the major factor in waterlogging stress is lack of O_2_, at least initially.

### 4.2. Effects on chlorophyll metabolism and photosynthesis

Chlorophyll is the predominant light-absorbing pigment for photosynthesis in plants. In grapevine, low-oxygen stress led to a significant inhibition in photosynthetic activities and a remarkable decrease in chlorophyll content (figure 1). The result was consistent with previous studies in which the waterlogging-induced chlorophyll loss was associated with the decrease of photosynthetic activities [48]. Further, transcriptomic data demonstrated that the application of exogenous waterlogging inhibited the activity of chlorophyll biosynthesis enzymes and induced the activity of chlorophyll degradation enzymes (figures 9*a* and 4; electronic supplementary material, table S4). The decline in chlorophyll content is believed to be due to acceleration in chlorophyll degradation and/or the blocking of chlorophyll synthesis.

Furthermore, most photosynthesis-related genes, involving PSII, PSI, cytochrome b6/f complex, ATP synthase, photosynthetic electron transport chain and photosynthesis–antenna proteins, were also significantly decreased (figure 9*a*; electronic supplementary material, table S4). Interestingly, 15 transcripts in PSII showed significant differential expression, while only 11 transcripts in PSI showed differential expression, suggesting that PSII was more sensitive than PSI under waterlogging stresses. The result was consistent with previous reports that PSI was usually more stable than PSII under stresses [49–51]. Altogether, these results at the transcriptomic and physiological level suggested that waterlogging stress in grapevine was undoubtedly closely tied to primary photosynthesis metabolic processes, and the decrease of photosynthetic activities was associated with chlorophyll loss.

### 4.3. Effects on carbon and energy metabolism

Plants initiate several responses to alleviate the harm of O_2_ deprivation during flooding or waterlogging periods. The hypoxic responses include the downregulation of a suite of energy-related and O_2_-consuming metabolic pathways [52]. Examples of such metabolic adaptations to hypoxia include the downregulation of storage metabolism [52], the shift from invertase to sucrose synthase routes of sucrose hydrolysis [53] and the inhibition of mitochondrial respiration [54].

In the leaves of waterlogged grapevine, many genes with potential roles in carbon and energy metabolism were identified as having significant transcriptional responses to stress. The most notable examples are the upregulation of genes involved in glycolysis and fermentation, and the downregulation of genes involved in sucrose and starch metabolism, citrate cycle, mitochondrial electron transport and photosynthesis (figures 9*b* and 5; electronic supplementary material, table S5). The comparisons of early transcriptomes of *Arabidopsis*, maize and cotton responses to waterlogging found that hypoxia triggers the overexpression of TPS in all three species [55–57]. TPS catalyses the first step of trehalose synthesis, which is important in plant response to abiotic stresses [58]. TPS has been shown to regulate sugar metabolism in plants [59], so the upregulation of TPS in the leaf indicated the acceleration of sugar metabolism in the grapevine leaf.

Most recent studies on carbon utilization for waterlogging tolerance responses focused on ADH in this complicated cycle of pathways. Indeed, the *ADH* gene was found to exhibit bidirectional functions in regulating waterlogging responses in the present study. It was not surprising that phenotypic analysis of overexpression of ADH in rice [60], *Arabidopsis* [61], cotton [62] and other species gave a different conclusion.

A comparative analysis between plant species of transcriptional responses to hypoxia found contrasting expression profiles between the tolerant and susceptible species for genes encoding components of the mitochondrial electron transport chain, with genes mainly upregulated in *Arabidopsis*, but downregulated in rice [63]. In CT leaves, the majority of genes involved in the mitochondrial electron transport were downregulated. Whether the mitochondrial electron transport chain transcript changes are related to plant waterlogging tolerance requires further demonstration. Overall, the results revealed that waterlogging promoted catabolism of carbohydrates and a switch from oxidative to anaerobic respiration in grape leaves.

### 4.4. Effects on amino acid metabolism

The comparative analysis of early transcriptome responses to low-oxygen environments in *Arabidopsis*, cotton and poplar found that amino acid metabolism changes were common in these three dicotyledonous species, although there was almost no overlap between their particular responses [55] (figures 9*c* and 6; electronic supplementary material, table S6). Waterlogging also led to rapid changes in the levels of amino acids in grapevine leaves. In CT, transcriptional downregulation of genes involved in glutamate degradation was found. However, large numbers of genes involved in aspartate degradation were upregulated.

As a result, a rapid increase in glutamate and decrease in aspartate may be found in the leaf. The same dynamic changes were found in the metabolite profiling of grey poplar root during hypoxia [9]. Kreuzwieser *et al*. proposed that hypoxia led to the inhibition of the TCA cycle and activation of glycolysis and fermentation pathways, resulting in an accumulation of amino acids closely derived from intermediates of glycolysis (e.g. glutamate) and a decrease of TCA cycle intermediate-derived amino acids (e.g. aspartate) [9].

We found that a lot of genes induced by waterlogging were related to protein degradation. The results implied that degradation of aerobic proteins would help decrease the consumption of oxygen, and abundant free amino acids should be derived at the same time. Free amino acids have two destinations, protein synthesis and amino metabolism. Hypoxia can repress overall protein synthesis in plants [64]. Accordingly, accumulated free amino acids from protein degradation should act as substrates in amino acid metabolism. It seems that in leaves of grapevine seedlings under waterlogging stress, amino acid metabolism has two main roles (figure 6): (a) generation of glutamate and alanine, which are critical for regulation of cytoplasmic pH; and (b) breakdown of carbon skeletons and generation of intermediates for energy metabolism.

Recently, most studies of carbon utilization for flooding tolerance response focused on PDC and ADH in this complicated cycle of pathways. It was not surprising that phenotypic analysis of overexpression of PDC or ADH in rice [60], *Arabidopsis* [61], cotton [62,65] and other species [66] has provided different conclusions. Based on the results in this study, genes involved in protein degradation and amino acid synthesis are ideal candidate genes that enhance flooding tolerance of plants along with *PDC*/*ADH*.

### 4.5. Effects on antioxidative defence system

Mustroph *et al*. compared transcriptomic adjustments to low-oxygen stress in 21 organisms across four kingdoms (Plantae, Animalia, Fungi and Bacteria) and found that the induction of enzymes and ROS generation/scavenging was a universal stress response found in the majority of the evaluated species and especially in all plants [67]. One of the major sources of ROS in plants is a reaction mediated by NAPDH oxidase, which is responsible for the conversion of O_2_ to superoxide anion (O^2−^), thereby leading to the production of hydrogen peroxide (H_2_O_2_).

To prevent the formation of ROS under stress, plants have evolved a complex antioxidative defence system: low molecular mass antioxidants (ascorbic acid, glutathione and tocopherols), enzymes regenerating the reduced forms of antioxidants and ROS-interacting enzymes such as SOD, POD and CAT [68]. Remarkably, the expression of alternative oxidase (AOX) was specifically enhanced in roots of waterlogged grapevine (VIT_02s0033g01400.t01, VIT_00s0399g00060.t01, VIT_02s0033g01380.t01) implying that AOX may function as an alternative to cytochrome oxidase under low-oxygen conditions. The increase in AOX gene expression could prevent ROS formation by the over-reduction of the ubiquinone pool. Based on the results, it is possible that the acclimation to waterlogging may not be dependent on SOD, POD and glutathione S-transferase, but on AOX, CAT and glutathione S-transferase (figures 9*d* and 7; electronic supplementary material, table S7).

Many antioxidant enzymes have been proved to be critical for the survival of many plants under different levels of waterlogging, e.g. rape [14], cucumber [12], winter wheat [69], citrus [70], soya bean [71] and *Mentha aquatic* [72]. In these plants, the expression patterns of three ROS-scavenging-related genes showed significant differential under flooded conditions, decreased expression of superoxide dismutase gene, and increased expression of peroxidase and catalase gene. Lee *et al*. showed downregulation of the *CAT* gene and upregulation of *POD*, *SOD* and *glutathione-S-transferase* in leaves of rape seedlings under waterlogging stress [14]. Qi *et al*. suggested that *POD* was upregulated, whereas *SOD*, *CAT* and *glutathione S-transferase* were downregulated under stress [12]. Consistent with numerous studies that have shown a correlation between the ability to ameliorate ROS and survival under different levels of waterlogging, the high induction of ROS network proteins in waterlogged grapevine showed that strong detoxification was critical for survival.

### 4.6. Effects on the signal transduction

Phytohormones, such as SA, ETH, JA and ABA, are crucial signalling molecules and play central roles in responses to abiotic and biotic stress signalling. For example, SA, JA and ETH have fundamental roles in biotic stress signalling. ABA is involved in the response to abiotic stress and appears to function as a negative regulator in disease resistance, in action opposite to that of SA, ETH and JA. Plant hormones are involved in several metabolic and development processes, and our data indicate the involvement of some of these hormones in plant growth in waterlogged soil. The results of the global grapevine transcriptome analysis also revealed the interactions between metabolism and signal transduction pathways of hormones and waterlogging stress in leaves of grapevine.

In our study, IAA, ETH, JA, ABA, BR and GA were identified in response to waterlogging stress. Most key enzymes for JA (LOX, JAZ and AOC), SA (PAL), ABA (ZEP, NCED and VDE) and ETH (ACO) biosynthesis and auxin/ETH-responsive transcription factor were significantly upregulated (figures 9*e* and 8; electronic supplementary material, table S8), suggesting that ETH, ABA and JA play a positive role as signalling molecules in response to waterlogging stress. However, most auxin signalling genes were downregulated, suggesting that auxin signalling genes may be negative regulators in waterlogging stress, in action opposite to that of ETH, ABA and JA. Further, BRs, the new class of steroidal hormones, confer tolerance in plants to cope with biotic and abiotic stress [73]. The sterol 24-C-methyltransferase, which is also essential for BR biosynthesis, was upregulated, possibly to facilitate the important role of BRs in the plant response to waterlogging stress. Additionally, all auxin influx carriers were downregulated and most AUX/IAA proteins and IAA synthase were upregulated by waterlogging stress in the IAA pathway. These observations imply that the IAA-regulated genes may play positive roles in plant development and abiotic stress responses. Furthermore, the relationship between IAA and oxidative stress tolerance will be interesting to investigate in the future.

ETH, ABA and SA were implicated as major hormones involved in the waterlogging response of grapevine. ETH entrapment by water represents the first warning signal to the plant indicating waterlogging, and auxin transport is stimulated by ETH [74]. In the light of the importance of hormone responses in the submergence phenotypes of rice [8], *Taxodium* [75] and hypoxia *Arabidopsis* [5–7], ERF, NCED and PAL in grapevine leaves are good candidate genes for the functional analysis of waterlogging tolerance.

### 4.7. Effects on non-symbiotic haemoglobins

Recent research by Narsai *et al*. [63,76] on comparative analysis between plant species of transcriptional and metabolic responses to hypoxia paid special attention to the possible relationship between haemoglobin expression and plant tolerance to low-oxygen conditions. The non-symbiotic haemoglobins are more commonly discussed in most plants [77]. Narsai *et al*. found that class 1 non-symbiotic haemoglobins rapidly increased under hypoxia in intolerant *Arabidopsis*, but were downregulated or unchanged in tolerant rice and poplar; genes encoding class 2 and class 3 haemoglobins also showed similar but less extreme trends [63,76]. Parent *et al*. proposed that non-symbiotic haemoglobins acted as a nitric oxide (NO) dioxygenase to convert NO to nitrate [45]. This pathway not only eliminated the toxic NO in the cell, but also helped maintain ATP synthesis. There were two genes (VIT_03s0063g01970.t01, VIT_03s0063g01960.t01) annotated as encoding class 2 non-symbiotic haemoglobin. According to the above hypothesis, this may facilitate grapevine waterlogging tolerance. Additionally, mRNAs related to haeme (i.e. non-symbiotic haemoglobin) binding were highly upregulated in the CT root. Thus, more attention needs to be paid to the relationship between non-symbiotic haemoglobins and grapevine waterlogging tolerance.

Low-oxygen conditions in plants promote the utilization of $\text{N}{{\text{O}}_{3}}^{-}$ and the production of NO to facilitate an aerobic survival [78,79]. However, apart from nitrate transporter genes being down- or upregulated, the expression changes in nitrate metabolism-related genes were not observed upon waterlogging in grapevine, including genes encoding nitrate reductase (a key enzyme responsible for conversion of nitrate to nitrite and NO) and nitrite reductase (an enzyme responsible for conversion of $\text{N}{{\text{O}}_{2}}^{-}$ to ammonium and to NO). These results suggest the regulation of nitrogen metabolism might be less important for waterlogging acclimation in grapevine.

### 4.8. The feasibility of using gene expression signals to predict the severity of damage caused to grapevine by waterlogging stress

Traditionally, the growth and development condition and the phenological period of crops are the main reference basis for agricultural production. This information is mainly collected through observing and recording the phenotypic trait of crops. This method has played an important role in traditional agricultural production that is intuitive, simple and effective. However, the appearance of phenotypic traits always lags, and cannot give feedback on the real growth and development situation of crops that is timely and accurate. What is worse, it is always too late to take remedial measures when the negative phenotype appears. Every trait of a plant is genetically controlled by genes, and the gene information can accurately reflect the real growth, development and metabolic status. Furthermore, the expression of gene is always earlier than the appearance of the phenotype, so monitoring and diagnosing the growth and development status of crops at the gene level make it possible to predict any unfavourable change or harm before it happens, and take remedial measures in advance.

The development of modern molecular biology techniques such as transcriptome sequencing technology and quantitative reverse transcription PCR provides extremely rich and even redundant gene information to researchers. Even though the gene expression profile has been widely used to address the relationship between ecologically influenced or disease phenotypes and the cellular expression patterns, the information is mainly limited to laboratory experience. It is necessary to apply the crop growth and development-related gene information to agricultural production, so the techniques or methods that are able to convert the gene information to convenient and easy access information are needed urgently. As predicted by Boss & Thomas [80], gene information is finding application in many aspects of practical production, meaning the era of applying gene information in practical fruit crop production is coming.

Recently, the development of molecular biotechnology, especially the development of one-step PCR, which is a simple, rapid and sensitive technique in detecting gene expression levels, makes it possible to monitor the gene expression status of the crops in real time. In this case, by detecting the expression levels of some marker genes corresponding to particular physiological activities, like drought response, high salinity response etc., we can quickly diagnose the impending drought or salt damage and thus take remedial measures in advance. In this study, to verify the feasibility of using gene expression signals to predict the severity of damage caused to grapevine by waterlogging stress, grapevine seedlings were treated with waterlogging for different time durations and then recovered by washing off the waterlogging. The results showed that grapevine seedlings can be rescued by washing off the waterlogging within 48 h of waterlogging stress. It is worth mentioning that similar expression patterns of VIT_14s0108g00540.t01, VIT_04s0044g00020.t01, and VIT_03s0038g04080.t01 were detected at this critical time (48 h). Thus, in turn, we can consider these expression patterns as the marker signals to predict the severity of salt stress damage. These three genes were involved in maintaining the structural and functional integrity of the plant cells. For example, gene VIT_14s0108g00540.t01 (phosphofructokinase, PFK) catalyses the phosphorylation of fructose-6-phosphate to fructose-1,6-bisphosphate, which is a key regulatory step in the glycolytic pathway and an important reaction in a wide variety of BPs. VIT_04s0044g00020.t01 (catalase, CAT) is a very important enzyme for protecting the cell from oxidative damage by ROS and VIT_03s0038g04080.t01 (glutamyl-tRNA synthetase, GLTX) plays a critical role in chlorophyll synthesis. It makes sense that their transcription status reflects the vitality of plants and thus can be used as marker signals to predict the damage severity caused by salt stress. In practical production, e.g. grape cultivation in regions with high rainfall, when genes VIT_04s0044g00020.t01 and VIT_14s0108g00540.t01 were detected, there was a sharp increase in expression signals and when VIT_03s0038g04080.t01 was detected, there was a sharp decrease in expression signals; this means that the grapevine seedlings have been damaged too much by waterlogging and will ultimately die. This technique can also be used in the prediction of severity of damage to crops caused by other agricultural disasters such as drought, salt, freezing etc. This will greatly benefit farmers especially in agricultural disaster-prone areas. To our knowledge, there are no reports to date on the use of gene expression information to predict whether plants are under stress, particularly in fruit crops.

## 5. Conclusion

Global warming results in extreme climates, such as drought, flooding and heat stress. Improvement of combined stress tolerance could benefit crop production. It is widely known that stresses, including drought, flooding and high temperatures, could trigger accumulation of ROS, which causes oxidative damage of CCs. On the other hand, ROS serve as critical signalling molecules in the oxidative stress response. This study identified numerous genes that are differentially expressed between control and waterlogging stress treatment, and provided an overview of waterlogging stress acclimation in grapevine. Genome-wide transcriptome analysis results indicated that waterlogging stress downregulated many genes related to chlorophyll synthesis and photosynthesis, and also upregulated many genes related to stress tolerance, including antioxidant defence systems, as well as the anaerobic fermentation and glycolysis-related genes.

In conclusion, the results indicate that genes related to many kinds of MFs were changed in grapevine leaf under waterlogged conditions. Complex interactions between chlorophyll metabolism, carbon metabolism, ROS generation or scavenging, and hormone signals of waterlogged grapevine leaf were observed. ETH and ABA were implicated as major hormones involved in the waterlogging response of grapevine. In light of the importance of mastering in advance the growth status of a plant under stress, three genes in grapevine leaf were found to be good candidate genes for predicting the grapevine growth status under waterlogging stress. There is also a challenge with an urgent need to find and characterize the complex interactions between waterlogging stress and defence in whole plants, with better assessment of agronomic practices and management to enhance plant growth and accelerate the progress in cultivating waterlogging-resistant plants for crop improvement.

## Supplementary Material

Real-time PCR primer information of 30 differently expressed genes.

## Supplementary Material

List of differentially regulated genes.

## Supplementary Material

List of genes only expressed in one library.

## Supplementary Material

Detail information of differential expressed genes related to chlorophyll metabolic and photosynthesis of grapevine under waterlogging stress.

## Supplementary Material

Detail information of differential expressed genes related to chlorophyll metabolic and photosynthesis of grapevine under waterlogging stress.

## Supplementary Material

Zhu_ tables _ESM 6.xlsx

## Supplementary Material

Zhu_ tables _ESM 7.xlsx

## Supplementary Material

Zhu_ tables _ESM 8.xlsx

## Supplementary Material

Zhu_ tables _ESM 9.xlsx

## Supplementary Material

Zhu_ figures _ESM 1.png

## Data accessibility

This transcriptome project has been deposited at NCBI SRA (Sequence Read Archive) database (http://www.ncbi.nlm.nih.gov/sra/). The BioProject ID related to this paper is SRP070475 (https://www.ncbi.nlm.nih.gov/sra/?term=SRP070475).
