# Supplementary figures and images for: Analysis of the regulation networks in grapevine reveals response to waterlogging stress and candidate gene-marker selection for damage severity
Source: R Soc Open Sci. 2018 Jun 27;5(6):172253. doi: 10.1098/rsos.172253 (PMC6030322; doi:10.1098/rsos.172253)

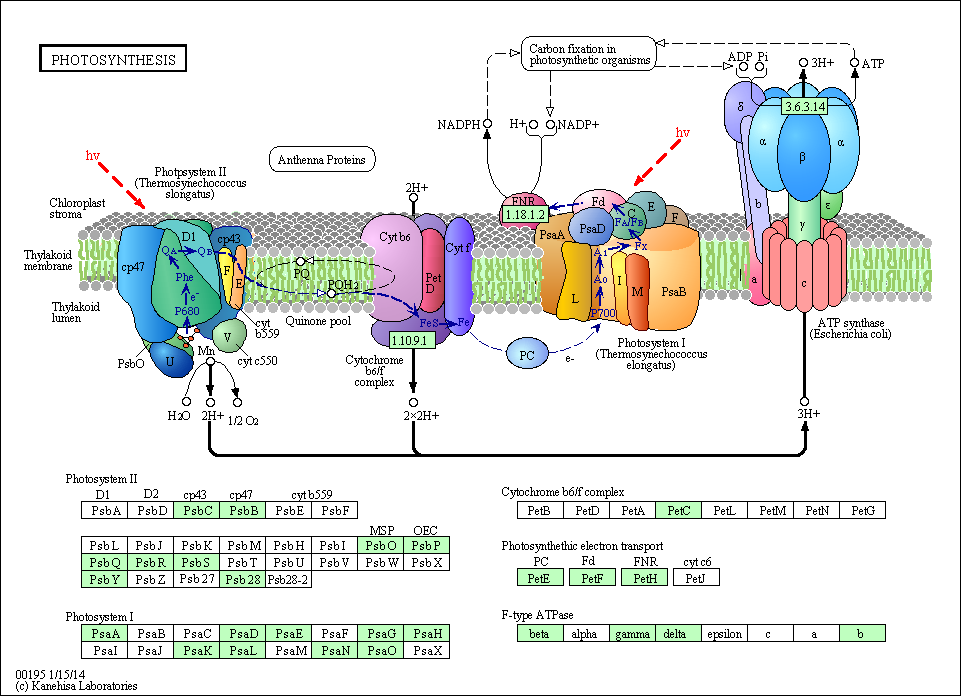

Supplement: Zhu_ figures _ESM 1.png [file rsos172253supp10.png]
